# The Evolving Role of Grit: Shifts in Depression Risk Among High- and Low-Grit Individuals During COVID-19

**DOI:** 10.3390/healthcare13070793

**Published:** 2025-04-02

**Authors:** Nicholas Lassi

**Affiliations:** School of Law, Southwest University of Political Science and Law, Chongqing 401120, China; luckynickphd@gmail.com

**Keywords:** grit, personality traits, depressive symptoms, mental health, public health crises

## Abstract

**Background/Objectives**: This study examined the relationship between grit—a measure of perseverance and diligence—and depressive symptoms in the context of the COVID-19 pandemic. While low levels of grit have typically been associated with increased vulnerability to mental health challenges, the pandemic introduced elements that may have altered this relationship. **Methods**: Using data from 5039 participants in the National Longitudinal Survey of Youth 97, one-way MANCOVAs were conducted to compare depressive symptoms across low-, moderate-, and high-grit individuals before and during the pandemic. **Results**: The findings show that in pre-pandemic periods, low-grit individuals displayed a significantly higher risk of depressive symptoms than their moderate- and high-grit counterparts. However, during the pandemic, this differential risk diminished, with higher-grit groups showing depressive symptoms comparable to those of lower-grit groups. **Conclusions**: These results suggest that public health crises entailing limited public mobility and social distancing may alter the traditional protective role of grit, prompting further investigation into how resilience factors interact with external stressors during times of widespread adversity.

## 1. Introduction

The COVID-19 pandemic, originating in late 2019, had an exceptional influence on mental health. According to the World Health Organization, global rates of anxiety and depression increased by an estimated 25 percent during the pandemic [1]. This rise in psychological distress was shaped by a variety of factors, with personality traits among the likely contributors to individual differences in mental health outcomes [2,3]. Examining how personality characteristics, specifically grit, may have moderated the psychological impact of the pandemic is valuable for developing effective mental health interventions and preventive strategies. Studying grit provides a view into how individuals sustain mental stability during exceptional public health crises.

Defined by the Cambridge Dictionary as “courage and determination despite difficulty” [4], grit encompasses hard work, diligence, and perseverance in facing challenges. Generally stable in adulthood, grit is commonly assessed using the Grit Scale (Grit-O) or the Short Grit Scale (Grit-S) [5,6,7]. Prior to the COVID-19 pandemic, studies had firmly established a relationship between grit and depression, providing a scaffold for interpreting the current study [8]. High levels of grit have been associated with a range of positive life outcomes [9,10,11,12], including employment success [10], academic achievement [13,14,15], and favorable psychological health [7,16]. High grit levels have consistently been linked with lower depression rates across diverse populations [17,18], such as university students and individuals managing chronic medical conditions [8,19]. Grit also appears to mitigate psychological distress [9,20], hopelessness, and suicidal thoughts [21,22]. This relationship may be bidirectional, with low grit levels increasing susceptibility to depression, and depression, in turn, diminishing grit.

However, evidence indicates that grit may not always be advantageous. For instance, studies have shown that grit was not linked with dropout rates among Dutch marine recruits during training [23], nor was it associated with specific characteristics of burnout, such as emotional exhaustion and cynicism, among internal medicine residents [24]. Furthermore, excessive grit, especially in work-related pursuits, may become detrimental if it limits time for leisure activities, which is important for reducing depression and promoting positive mental health [25,26].

The public health restrictions implemented during the COVID-19 pandemic—such as lockdowns, social distancing, and limitations on work and daily activities—brought about unprecedented social challenges, fundamentally altering daily routines, social interactions, and economic productivity. Unlike under normal conditions, where persistence and the extended drive to achieve goals are beneficial, the pandemic produced conditions that may have made these characteristics less useful or even unproductive. 

While prior research has analyzed grit’s relationship with psychological well-being under typical conditions [17,18], relatively limited investigations have been conducted on the significant and extensive stressors presented by a global pandemic, and the existing studies in this context have produced mixed findings. For example, Toczko et al. determined that grit was not significantly linked with depression during the initial phases of the COVID-19 pandemic [27]. However, a study by Liu et al. specified that higher levels of grit were associated with lower levels of depression amid the pandemic, stressing the possible protective influence of grit during these exceptional circumstances [28]. Similarly, Akaishi et al. found that each one-unit increase in the Grit-S score during the pandemic corresponded to a 63 percent decrease in the likelihood of experiencing depressive symptoms [29]. These inconsistent findings show the need for additional investigations to better clarify grit’s relationship with mental well-being during prolonged crises. The present study addresses this gap by examining the relationship between varying grit levels and depressive symptoms before and during the COVID-19 pandemic.

This study investigates how an international public health crisis like COVID-19 may differentially impact mental health based on individuals’ levels of grit. Derived from the existing literature and the purpose of this research, the following hypotheses are proposed:

1. Prior to the COVID-19 pandemic, individuals with more grit will display significantly lower levels of depressive symptoms relative to individuals with less grit.
2. During the COVID-19 pandemic, the divide in depressive symptoms between high-grit and low-grit individuals will tighten, with high-grit individuals undergoing a greater rise in depressive symptoms, making their levels of depression more similar to those with low levels of grit.
3. The two elements of grit in this study (hard work and diligence) will have dissimilar connections with various depressive symptoms. For example, hard work may be more strongly linked with insusceptibility to depressive symptoms than diligence.

## 2. Materials and Methods

This study examined the relationship between grit and depressive symptoms before and during the COVID-19 pandemic through a retrospective observational analysis using secondary data from the National Longitudinal Survey of Youth 97 (NLSY97) dataset. Statistical analyses were conducted using one-way multivariate analysis of covariance (MANCOVA) to evaluate this relationship. The study focused on two grit variables from 2013: hard work and diligence. They were examined in relation to seven depression-related outcome variables collected in 2019 and 2021: (1) feeling depressed; (2) difficulty getting going; (3) restless sleep; (4) perceived effort in activities; (5) sadness; (6) trouble focusing; (7) poor appetite.

The NLSY97, funded by the U.S. Bureau of Labor Statistics, is a longitudinal study that examines labor market conditions, social experiences, and psychological well-being among people living in the U.S. Initiated in 1997, the NLSY97 cohort is currently being surveyed biennially and has completed 20 waves as of 2025. For the present study, the dataset consisted of 5039 individuals born between 1980 and 1984, representing both metropolitan and non-metropolitan areas. The sample is demographically representative, with 51.9 percent non-Black/non-Hispanic, 26 percent Black/non-Hispanic, 21.2 percent Hispanic, and 0.9 percent mixed-race respondents, as well as 51 percent male and 49 percent female [30]. The initial sample of 5616 subjects was adjusted to 5039 through listwise deletion to address missing data. During the 2021 data collection period, the participants were between the ages of 36 and 42 (mean age: 39). National Longitudinal Survey (NLS) interviewers conducted surveys via face-to-face, telephone, and computerized/online interviews (the predominant method), guaranteeing consistency and regularity in the assessments over time.

### 2.1. Variables Under Study

Independent variables: Participants responded to two grit-related questions from Duckworth et al.’s Grit Scale [6], measuring perceived levels of grittiness and perseverance:

1. Hard work: “I am a hard worker”. (Response options: 1 = Very much like me; 2 = Mostly like me; 3 = Somewhat like me; 4 = Not much like me; 5 = Not like me at all)
2. Diligence: “I am diligent”. (Response options: 1 = Very much like me; 2 = Mostly like me; 3 = Somewhat like me; 4 = Not much like me; 5 = Not like me at all)

Dependent variables: Seven depression-related questions were derived from the Center for Epidemiologic Studies Depression Scale (CES-D) [31]. Respondents rated each item based on their experiences during the week preceding the interview.

1. Feeling Depressed: “During the past week, I felt depressed”.
2. Difficulty “Getting Going”: “During the past week, I could not “get going”.
3. Restless Sleep: “During the past week, my sleep was restless”.
4. Effort in Activities: “During the past week, I felt everything I did was an effort”.
5. Feeling Sad: “During the past week, I felt sad”.
6. Trouble Focusing: “During the past week, I had trouble keeping my mind on what I was doing”.
7. Poor Appetite: “During the past week, I did not feel like eating; my appetite was poor”.

Responses for each question were rated as follows: 0 = Rarely/None of the time/0–1 day; 1 = Some/A little of the time/2 days; 2 = Occasionally/Moderate amount of the time/3–4 days; 3 = Most/All of the time/5–7 days.

### 2.2. MANCOVA Testing

Fourteen MANCOVAs were conducted to explore the association between grit and depressive symptoms, each addressing one of the following relationships:

1. Hard work and feeling depressed (2019 vs. 2021).
2. Hard work and difficulty “getting going” (2019 vs. 2021).
3. Hard work and restless sleep (2019 vs. 2021).
4. Hard work and effort in activities (2019 vs. 2021).
5. Hard work and feeling sad (2019 vs. 2021).
6. Hard work and trouble focusing (2019 vs. 2021).
7. Hard work and poor appetite (2019 vs. 2021).
8. Diligence and feeling depressed (2019 vs. 2021).
9. Diligence and difficulty “getting going” (2019 vs. 2021).
10. Diligence and restless sleep (2019 vs. 2021).
11. Diligence and effort in activities (2019 vs. 2021).
12. Diligence and feeling sad (2019 vs. 2021).
13. Diligence and trouble focusing (2019 vs. 2021).
14. Diligence and poor appetite (2019 vs. 2021).

Each analysis controlled for birth year, gender, and ethnicity to mitigate confounding variables. No outliers were removed from the dataset, as there was no indication of erroneous data. Missing data were managed through listwise deletion. To account for violations of multivariate normality and homogeneity of variance–covariance matrices, Pillai’s trace was employed in the MANCOVAs, as it is considered the most robust statistic under these circumstances. Bonferroni corrections were applied to the analyses to attend to the issue of multiple comparisons and decrease the potential for Type I errors.

## 3. Results

Test 1: Working hard and feeling depressed before (2019) and during (2021) the pandemic.

A significant multivariate test result was obtained from examining the relationship between working hard and feeling depressed in 2019 and 2021 (Pillai’s Trace = 0.012; F (8, 10,062) = 7.443; *p* < 0.001; η2p = 0.006; pwr = 1.00).

### 3.1. Working Hard and Feeling Depressed in 2019

Univariate testing indicated a significant difference between working hard and feeling depressed in 2019 (range: 0 to 3 (higher scores indicate more incidents of feeling depressed); F (4, 5031) = 11.748; *p* < 0.001; η2p = 0.009; pwr = 1.00). Post hoc comparisons using Bonferroni correction indicated significant differences in hard work, whereby those who responded with “not like me at all to work hard” (M = 0.96) had significantly higher levels of feeling depressed than those with “very much like me to work hard” (M = 0.26), “mostly like me to work hard” (M = 0.29), and “somewhat like me to work hard” (M = 0.44) responses (Table 1).

**Figure 1:**
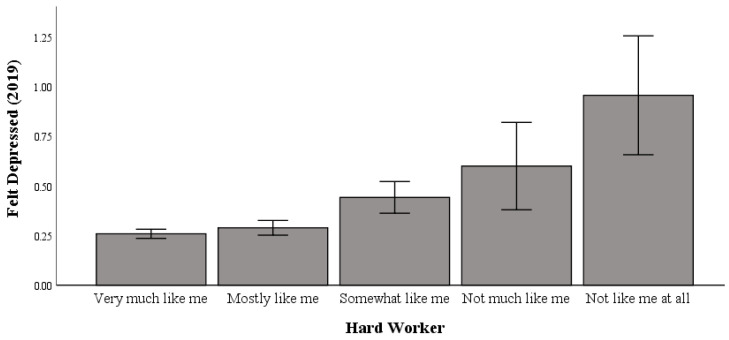
The relationship between working hard and feeling depressed in 2019.

As shown in Figure 1, in 2019, those most averse to hard work were linked to higher levels of feeling depressed relative to hard workers.

### 3.2. Working Hard and Feeling Depressed in 2021

Univariate testing indicated a significant difference between working hard and feeling depressed in 2021 (F (4, 5031) = 5.517; *p* < 0.001; η2p = 0.004; pwr = 0.98). Post hoc comparisons using Bonferroni correction indicated no significant differences between the “not like me at all” (M = 0.69) group and all other groups.

**Figure 2:**
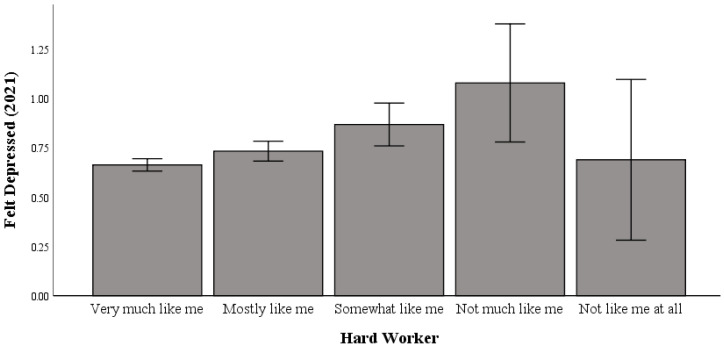
The relationship between working hard and feeling depressed in 2021.

As shown in Figure 2, “not like me at all” in working hard was not linked to higher levels of feeling depressed in 2021. This indicates that in 2021, compared with 2019, those most averse to hard work were more equivalent to hard workers in feeling depressed.

Test 2: Working hard and difficulty “getting going” before (2019) and during (2021) the pandemic.

A significant multivariate test result was obtained from examining the relationship between working hard and difficulty “getting going” in 2019 and 2021 (Pillai’s Trace = 0.013; F (8, 10,064) = 8.515; *p* < 0.001; η2p = 0.007; pwr = 1.00).

### 3.3. Working Hard and Difficulty “Getting Going” in 2019

Univariate testing indicated a significant difference in the relationship between working hard and “getting going” in 2019 (range: 0 to 3 (higher scores indicate more incidents of being unable to “get going”); F (4, 5032) = 10.931; *p* < 0.001; η2p = 0.009; pwr = 1.00). Post hoc comparisons using Bonferroni correction indicated significant differences in working hard, whereby the “not like me at all to work hard” (M = 0.75) group had significantly higher levels of difficulty “getting going” than the “very much like me to work hard” (M = 0.24) and “mostly like me to work hard” (M = 0.28) groups (Table 2).

**Figure 3:**
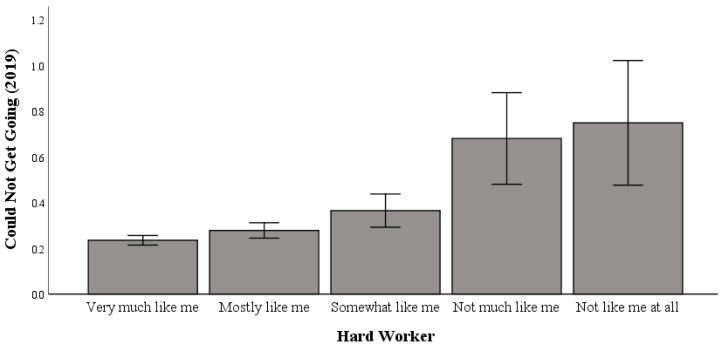
The relationship between working hard and difficulty “getting going” in 2019.

As shown in Figure 3, in 2019, those most averse to hard work were linked to higher levels of difficulty “getting going” relative to hard workers.

### 3.4. Working Hard and Difficulty “Getting Going” in 2021

Univariate testing indicated a significant difference between working hard and difficulty “getting going” in 2021 (F (4, 5032) = 9.203; *p* < 0.001; η2p = 0.007; pwr = 1.00). Post hoc comparisons using Bonferroni correction indicated no significant differences between the “not like me at all to work hard” (M = 0.491) groups and the “very much like me to work hard” (M = 0.62), “mostly like me to work hard” (M = 0.68), and “somewhat like me to work hard” (M = 0.84) groups in relation to difficulty “getting going”.

**Figure 4:**
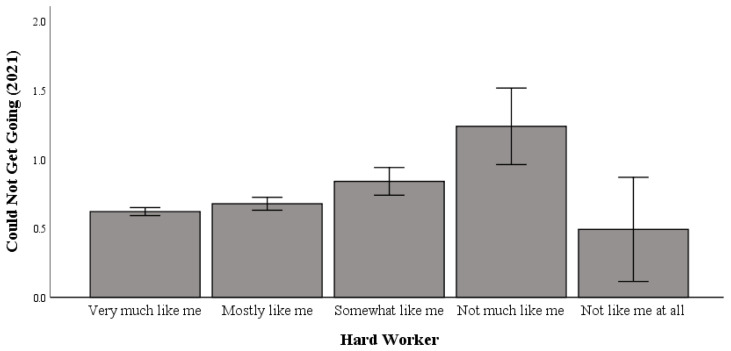
The relationship between working hard and difficulty “getting going” in 2021.

As shown in Figure 4, “not like me at all to work hard” was not linked to higher levels of difficulty “getting going” in 2021. This indicates that in 2021, compared with 2019, those most averse to hard work were more equivalent to hard workers in difficulty “getting going”.

Test 3: Working hard and restless sleep before (2019) and during (2021) the pandemic.

A significant multivariate test result was not obtained for the relationship between working hard and restless sleep (Pillai’s Trace = 0.003; F (8, 10,068) = 1.831; *p* = 0.067; η2p = 0.001; pwr = 0.79). Thus, no further tests were conducted.

Test 4: Working hard and feeling everything is an effort before (2019) and during (2021) the pandemic.

A significant multivariate test result was obtained for the relationship between working hard and feeling everything is an effort in 2019 and 2021 (Pillai’s Trace = 0.006; F (8, 9876) = 3.542; *p* < 0.001; η2p = 0.003; pwr = 0.98).

### 3.5. Working Hard and Everything Is an Effort in 2019

Univariate testing indicated a significant difference in the relationship between working hard and everything is an effort in 2019 (range: 0 to 3 (higher responses indicate elevated levels of everything being an effort); F (4, 4938) = 4.673; *p* = 0.001; η2p = 0.004; pwr = 0.95). Post hoc comparisons using Bonferroni correction indicated significant differences in working hard, whereby the “not like me at all to work hard” (M = 1.36) group had significantly higher levels of everything being an effort than the “very much like me to work hard” (M = 0.67) and “mostly like me to work hard” (M = 0.63) groups.

### 3.6. Working Hard and Everything Is an Effort in 2021

Univariate testing indicated a significant difference in the relationship between working hard and everything is an effort in 2021 (F (4, 4938) = 4.063; *p* = 0.003; η2p = 0.003; pwr = 0.92). Post hoc comparisons using Bonferroni correction indicated no significant differences between the “not like me at all to work hard” (M = 1.430) group and all the other groups in everything being an effort. This indicates that in 2021, compared with 2019, those most averse to hard work were more equivalent to hard workers in everything being an effort.

Test 5: Working hard and feeling sad before (2019) and during (2021) the pandemic.

A significant multivariate test result was obtained from examining the relationship between working hard and feeling sad in 2019 and 2021 (Pillai’s Trace = 0.009; F (8, 10,076) = 5.502; *p* < 0.001; η2p = 0.004; pwr = 1.00).

### 3.7. Working Hard and Feeling Sad in 2019

Univariate testing indicated a significant difference in the relationship between working hard and feeling sad in 2019 (range: 0 to 3 (higher scores indicate more incidents of feeling sad); F (4, 5038) = 8.816; *p* < 0.001; η2p = 0.007; pwr = 0.99). Post hoc comparisons using Bonferroni correction indicated significant differences in working hard, whereby the “not like me at all to work hard” (M = 0.76) group had significantly higher levels of feeling sad than the “very much like me to work hard” (M = 0.31) group.

### 3.8. Working Hard and Feeling Sad in 2021

Univariate testing indicated a significant difference in the relationship between working hard and feeling sad in 2021 (F (4, 5038) = 4.830; *p* = 0.001; η2p = 0.004; pwr = 0.96). Post hoc comparisons using Bonferroni correction indicated no significant differences between the “not like me at all to work hard” (M = 0.67) group and all the other groups in feeling sad. This indicates that relative to 2019, in 2021, those most averse to working hard were more equivalent to hard workers in feeling sad.

Test 6: Working hard and keeping the mind focused before (2019) and during (2021) the pandemic.

A significant multivariate test result was obtained from examining the relationship between working hard and keeping the mind focused in 2019 and 2021 (Pillai’s Trace = 0.020; F (8, 10,044) = 12.446; *p* < 0.001; η2p = 0.01; pwr = 1.00).

### 3.9. Working Hard and Keeping the Mind Focused in 2019

Univariate testing indicated a significant difference between working hard and keeping the mind focused in 2019 (range: 0 to 3 (higher responses indicate more incidents of being unable to keep the mind focused); F (4, 5022) = 19.095; *p* < 0.001; η2p = 0.015; pwr = 1.00). Post hoc comparisons using Bonferroni correction indicated significant differences in working hard, whereby the “not like me at all to work hard” (M = 1.59) group had significantly higher levels of being unable to keep the mind focused than the “very much like me to work hard” (M = 0.41), “mostly like me to work hard” (M = 0.47), “somewhat like me to work hard” (M = 0.68), and “not much like me to work hard” (M = 0.81) groups.

### 3.10. Working Hard and Being Unable to Keep the Mind Focused in 2021

Univariate testing indicated a significant difference between working hard and being unable to keep the mind focused in 2021 (F (4, 5022) = 10.436; *p* < 0.001; η2p = 0.008; pwr = 1.00). Post hoc comparisons using Bonferroni correction indicated no significant differences between the “not like me at all to work hard” (M = 1.11) group and all the other groups in keeping the mind focused. This suggests that in 2021, compared with 2019, those most averse to hard work were more equivalent to hard workers in being unable to keep the mind focused.

Test 7: Working hard and poor appetite before (2019) and during (2021) the pandemic.

A significant multivariate test result was obtained from examining the relationship between working hard and poor appetite in 2019 and 2021 (Pillai’s Trace = 0.008; F (8, 9976) = 4.966; *p* < 0.001; η2p = 0.004; pwr = 0.99).

### 3.11. Working Hard and Poor Appetite in 2019

Univariate testing indicated a significant difference in the relationship between working hard and poor appetite in 2019 (range: 0 to 3 (higher scores indicate elevated levels of poor appetite); F (4, 4988) = 7.898; *p* < 0.001; η2p = 0.006; pwr = 0.99). Post hoc comparisons using Bonferroni correction indicated significant differences in working hard, whereby the “not like me at all to work hard” (M = 0.73) group had significantly higher levels of poor appetite than the “very much like me to work hard” (M = 0.18), “mostly like me to work hard” (M = 0.20), and “somewhat like me to work hard” (M = 0.26) groups.

### 3.12. Working Hard and Poor Appetite in 2021

Univariate testing indicated a significant difference in the relationship between working hard and poor appetite in 2021 (F (4, 4988) = 3.703; *p* = 0.005; η2p = 0.003; pwr = 0.89). Post hoc comparisons using Bonferroni correction indicated no significant differences between the “not like me at all to work hard” (M = 0.63) group and all the other groups in poor appetite. This indicates that in 2021, compared with 2019, those most averse to hard work were more equivalent to hard workers in poor appetite.

Test 8: Diligence and feeling depressed before (2019) and during (2021) the pandemic.

A significant multivariate test result was obtained from examining the relationship between diligence and feeling depressed in 2019 and 2021 (Pillai’s Trace = 0.011; F (8, 9608) = 6.778; *p* < 0.001; η2p = 0.006; pwr = 1.00).

### 3.13. Diligence and Feeling Depressed in 2019

Univariate testing indicated a significant difference in the relationship between diligence and feeling depressed in 2019 (range: 0 to 3 (higher scores indicate more incidents of feeling depressed); F (4, 4804) = 10.978; *p* < 0.001; η2p = 0.009; pwr = 1.00). Post hoc comparisons using Bonferroni correction indicated significant differences in diligence, whereby the “not like me at all” (M = 0.54) group had significantly higher levels of feeling depressed than the “very much like me” (M = 0.22) and “mostly like me” (M = 0.27) groups.

### 3.14. Diligence and Feeling Depressed in 2021

Univariate testing indicated a significant difference in the relationship between diligence and feeling depressed in 2021 (F (4, 4804) = 5.069; *p* < 0.001; η2p = 0.004; pwr = 0.97). Post hoc comparisons using Bonferroni correction indicated no significant differences between the “not like me at all to be diligent” (M = 0.75) group and all other groups. This suggests that in 2021, compared with 2019, the low-diligence group was more equivalent to the diligent groups in feeling depressed.

Test 9: Diligence and difficulty “getting going” before (2019) and during (2021) the pandemic.

A significant multivariate test result was obtained from examining the relationship between diligence and being unable to “get going” in 2019 and 2021 (Pillai’s Trace = 0.017; F (8, 9622) = 10.461; *p* < 0.001; η2p = 0.009; pwr = 1.00).

### 3.15. Diligence and Being Unable to “Get Going” in 2019

Univariate testing indicated a significant difference in the relationship between diligence and being unable to “get going” in 2019 (range: 0 to 3 (higher responses indicate more incidents of being unable to “get going”); F (4, 4811) = 18.539; *p* < 0.001; η2p = 0.015; pwr = 1.00). Post hoc comparisons using Bonferroni correction indicated significant differences in diligence, whereby the “not like me at all” (M = 0.51) group had significantly higher levels of being unable to “get going” than the “very much like me” (M = 0.18), “mostly like me” (M = 0.25), and “not much like me” (M = 0.29) groups.

### 3.16. Diligence and Being Unable to “Get Going” in 2021

Univariate testing indicated a significant difference in the relationship between diligence and being unable to “get going” in 2021 (F (4, 4811) = 5.281; *p* < 0.001; η2p = 0.004; pwr = 0.97). Post hoc comparisons using Bonferroni correction indicated no significant differences between the “not like me at all to be diligent” (M = 0.60) group and all the other groups in being unable to “get going.” This indicates that in 2021, compared with 2019, the low-diligence group was more equivalent to the diligent groups in being unable to “get going”.

Test 10: Diligence and restless sleep before (2019) and during (2021) the pandemic.

A significant multivariate test result was obtained from examining the relationship between diligence and restless sleep (Pillai’s Trace = 0.004; F (8, 9622) = 2.390; *p* = 0.014; η2p = 0.002; pwr = 0.90). However, there was a limited relationship between diligence and restless sleep and no meaningful differences between 2019 and 2021.

Test 11: Diligence and feeling everything is an effort before (2019) and during (2021) the pandemic.

A significant multivariate test result was obtained from examining the relationship between diligence and feeling everything is an effort in 2019 and 2021 (Pillai’s Trace = 0.009; F (8, 9454) = 5.193; *p* < 0.001; η2p = 0.004; pwr = 0.99).

### 3.17. Diligence and Everything Is an Effort in 2019

Univariate testing indicated a significant difference in the relationship between diligence and everything is an effort in 2019 (range: 0 to 3 (higher responses indicate elevated levels of everything being an effort); F (4, 4727) = 9.192; *p* < 0.001; η2p = 0.008; pwr = 1.00). Post hoc comparisons using Bonferroni correction indicated significant differences in diligence, whereby the “not like me at all” (M = 0.94) group had significantly higher levels of everything being an effort than the “very much like me” (M = 0.57) group.

### 3.18. Diligence and Everything Is an Effort in 2021

Univariate testing indicated a significant difference in the relationship between diligence and everything is an effort in 2021 (F (4, 4727) = 2.373; *p* = 0.050; η2p = 0.002; pwr = 0.69). Post hoc comparisons using Bonferroni correction indicated no significant differences between the “not like me at all to be diligent” (M = 1.08) group and all the other groups in everything being an effort. This indicates that in 2021, compared with 2019, the low-diligence group was more equivalent to the diligent groups in everything being an effort.

Test 12: Diligence and feeling sad before (2019) and during (2021) the pandemic.

A significant multivariate test result was obtained from examining the relationship between diligence and feeling sad in 2019 and 2021 (Pillai’s Trace = 0.007; F (8, 9620) = 4.418; *p* < 0.001; η2p = 0.004; pwr = 0.99).

### 3.19. Diligence and Feeling Sad in 2019

Univariate testing indicated a significant difference in the relationship between diligence and feeling sad in 2019 (range: 0 to 3 (higher scores indicate more incidents of feeling sad); F (4, 4810) = 5.829; *p* < 0.001; η2p = 0.005; pwr = 0.98). Post hoc comparisons using Bonferroni correction indicated significant differences in diligence, whereby the “not like me at all” (M = 0.50) group had significantly higher levels of feeling sad than the “very much like me” (M = 0.28) group.

### 3.20. Diligence and Feeling Sad in 2021

Univariate testing indicated a significant difference in the relationship between diligence and feeling sad in 2021 (F (4, 4810) = 3.736; *p* = 0.005; η2p = 0.003; pwr = 0.89). Post hoc comparisons using Bonferroni correction indicated no significant differences between the “not like me at all to be diligent” (M = 0.71) group and all the other groups in feeling sad. This indicates that in 2021, compared with 2019, the low-diligence group was more equivalent to the diligent groups in feeling sad.

Test 13: Diligence and keeping the mind focused before (2019) and during (2021) the pandemic.

A significant multivariate test result was obtained from examining the relationship between diligence and keeping the mind focused in 2019 and 2021 (Pillai’s Trace = 0.011; F (8, 9598) = 6.577; *p* < 0.001; η2p = 0.005; pwr = 1.00). However, there was a limited relationship between diligence and keeping the mind focused and no meaningful differences between 2019 and 2021.

Test 14: Diligence and poor appetite before (2019) and during (2021) the pandemic.

A significant multivariate test result was obtained from examining the relationship between diligence and poor appetite in 2019 and 2021 (Pillai’s Trace = 0.005; F (8, 9544) = 2.906; *p* = 0.003; η2p = 0.002; pwr = 0.96). However, there was a limited relationship between diligence and poor appetite and no meaningful differences between 2019 and 2021.

## 4. Discussion

This study examined the relationship between grit and depressive symptoms prior to and during the COVID-19 pandemic. Before the pandemic, the data indicated that individuals with low levels of grit exhibited a higher propensity for depressive symptoms compared to those with moderate and high levels of grit. However, a notable shift in this relationship was observed during the pandemic, as depressive symptoms increased more significantly among high- and moderate-grit individuals than among low-grit individuals. Given that low-grit individuals showed more vulnerability to depressive symptoms before the pandemic, the likelihood of experiencing depressive symptoms became more comparable across low and high/moderately high-grit individuals during COVID-19. The findings also showed no notable differences between the two elements of grit (hard work and diligence) in their relationship with different depressive symptoms—indicating that both items operate similarly with respect to depression. These results suggest that the pandemic altered the relationship between grit and psychological health, showing the need for further investigation into how external crises reshape psychological well-being across various levels of grit.

Work responsibilities were altered during the pandemic, with many individuals experiencing reduced workloads or ceasing work altogether. Lockdowns and stay-at-home mandates confined people to their homes, distancing them from workplaces and routine duties. This transition may have exerted less psychological strain on individuals less inclined toward high work engagement. These individuals may have adapted more easily to a new environment characterized by limited work and responsibilities. Characteristics often associated with lower levels of grit, such as a more sedentary lifestyle and a lower propensity for work intensity [32], align well with the conditions imposed by lockdowns, social distancing, and other public health controls.

Those predisposed to low levels of grit may have experienced a greater sense of social acceptance regarding a more relaxed work pace. These individuals might have observed a collective sentiment of understanding and endorsement for reduced workloads, providing affirmation that a less-demanding lifestyle is acceptable or preferable. This validation may have increased a sense of contentment among low-grit individuals, contributing to relative stability in their depressive symptoms during the pandemic.

Conversely, individuals with high levels of grit may have struggled with the widespread reduction in productivity. The perception of falling behind due to mandated inactivity could have been particularly distressing for these individuals. Disruptions to their routines and constraints on their usual productivity may have elevated depressive symptoms in those accustomed to a high level of work engagement.

These results correspond with Hobfoll’s Conservation of Resources (COR) theory, which postulates that individuals endeavor to attain, protect, and build resources under strain [33,34]. The stress response centers on minimizing losses and optimizing gains, with the prevention of losses outweighing the pursuit of further gains [35]. Loss of resources is a powerful predictor of the intensity and persistence of mental distress following natural disasters or extended military combat [35,36,37]. In the face of stay-at-home orders and other prolonged work disruptions, individuals with high grit levels were often unable to harness this resource-preserving response, possibly contributing to depressive symptoms.

While diminished work engagement may partly account for the heightened depressive symptoms detected among high-grit individuals during COVID-19, reduced social support and augmented family responsibilities, such as supervising the remote education of children, may have produced added challenges that advanced depressive symptoms.

### Limitations

This study’s reliance on self-reporting measures to evaluate grit and depressive symptoms introduces potential biases, such as respondents altering their responses in pursuit of social acceptance and inaccuracies in self-evaluation. These biases may lead individuals to overestimate or underestimate the real extent of their grit and depression, which could strengthen or diminish the relationship detected. The subjective nature of self-assessments may, therefore, influence the validity and accuracy of the conclusions. Future studies might include more objective evaluations—such as observing behavioral conduct or third-party assessments—to support self-evaluation data.

The existing literature indicates that grit and conscientiousness are largely stable over time (e.g., in test–retest stability over extended timeframes), particularly after the early phases of life [5,38,39,40]. Given that grit was assessed in 2013, when the sample consisted of participants in their late 20s to early 30s, it is fair to surmise that grit levels were fairly consistent during the period prior to the evaluations of depressive symptoms in 2019 and 2021. Nonetheless, it is recognized that unobserved variability in grit over time could have affected the results.

The timeline of the COVID-19 pandemic complicates conclusions derived from the observed levels of depression. This timeline includes the onset and cessation of public health controls and social and medical policy shifts. Variations in vaccine availability, recurrent waves of infection, and fluctuating stay-at-home mandates may have contributed to changing rates of depression, adding complexity in pinpointing depressive symptoms to specific points in time during the COVID-19 pandemic.

While the tests in the current study controlled for birth year, ethnicity, and gender, it is recognized that other variables—such as socioeconomic status (SES), access to mental health care, or susceptibility to long COVID—may affect the results [41]. The absence of these and related covariates is a limitation. Additionally, the assessment of grit in the current study was obtained from two items of Duckworth et al.’s scale [6], which evaluates the central features of grit, including persistence and diligence. However, grit is understood to be a multi-component concept, consisting of determination and consistency of interests throughout extended timeframes. Depending exclusively on these two elements may not wholly encapsulate the totality of grit, possibly overlooking other aspects that could affect its relationship with depressive symptoms. Future studies should consider more expanded evaluations, such as the complete Grit-O or Grit-S measures, and potentially employ other assessment instruments (e.g., behavioral evaluations) to offer a more thorough measure of grit.

Although the findings are statistically significant, the effect sizes are modest and should be considered within a practical context. The magnitude of the association between grit and depressive symptoms may not be adequate to independently produce meaningful progress in mental health outcomes. Future studies might evaluate whether interventions seeking to improve grit can generate significant and clinically relevant advances in mental health. Also, given the study’s design, causality cannot be established, and the observed directional effects remain unclear.

To the best of the author’s knowledge, this study is the first to identify this phenomenon. Although it provides an initial basis for discourse, it also shows a need for careful interpretation and a cautious approach to generalization. Future research should explore the specific mechanisms through which grit is associated with psychological health during crises and seek to develop interventions to support individuals with varying levels of grit during such periods. Furthermore, extended longitudinal research studies could offer a deeper understanding of how grit is associated with long-term psychological health post-crisis.

## 5. Conclusions

This study assessed the relationship between grit and depressive symptoms, detailing how the COVID-19 pandemic reshaped this relationship. Prior to the pandemic, individuals with lower levels of grit exhibited a higher risk for depressive symptoms compared with those with moderate or high grit levels. However, during the pandemic, this relationship shifted, with individuals of higher grit experiencing a more pronounced increase in depressive symptoms relative to those with low grit. This change suggests that the pandemic imposed unique psychological challenges on those with stronger perseverance. Recognizing these shifts in depressive symptoms across different grit levels can help guide more personalized mental health interventions during times of crisis. These findings may also serve as a valuable resource for individuals experiencing other extended crises, such as during economic declines.

## Figures and Tables

**Table 1:** Mean, std. error, and 95% confidence interval (CI) for working hard and feeling depressed.

Dependent Variable	Hard Worker	Mean	Std. Error	95% CI	
				Low Bound	Up Bound
Felt depressed (2019)	Very much like me	0.258	0.012	0.235	0.281
	Mostly like me	0.289	0.019	0.251	0.326
	Somewhat like me	0.442	0.041	0.362	0.521
	Not much like me	0.599	0.112	0.379	0.819
	Not like me at all	0.955	0.153	0.656	1.255
Felt depressed (2021)	Very much like me	0.662	0.016	0.631	0.693
	Mostly like me	0.732	0.026	0.681	0.782
	Somewhat like me	0.867	0.055	0.758	0.975
	Not much like me	1.077	0.153	0.778	1.376
	Not like me at all	0.688	0.208	0.281	1.095

**Table 2:** Mean, std. error, and 95% CI for working hard and difficulty “getting going”.

Dependent Variable	Hard Worker	Mean	Std. Error	95% CI	
				Low Bound	Up Bound
Could not “get going” (2019)	Very much like me	0.235	0.011	0.214	0.256
	Mostly like me	0.278	0.017	0.244	0.312
	Somewhat like me	0.365	0.037	0.292	0.437
	Not much like me	0.680	0.102	0.480	0.880
	Not like me at all	0.748	0.139	0.476	1.020
Could not “get going” (2021)	Very much like me	0.620	0.015	0.591	0.649
	Mostly like me	0.677	0.024	0.630	0.723
	Somewhat like me	0.839	0.051	0.738	0.939
	Not much like me	1.237	0.141	0.960	1.514
	Not like me at all	0.491	0.192	0.114	0.868

## Data Availability

The data used in this study are available from the National Longitudinal Survey of Youth 97: https://www.nlsinfo.org/content/cohorts/nlsy97 (accessed on 30 March 2025).
